# Synthesis and Application of Reactive Acrylic Latexes: Effect of Particle Morphology

**DOI:** 10.3390/polym14112187

**Published:** 2022-05-27

**Authors:** Catalina N. Cheaburu-Yilmaz, Cigdem Kilicarislan Ozkan, Onur Yilmaz

**Affiliations:** 1 Laboratory of Physical Chemistry of Polymers, Petru Poni Institute of Macromolecular Chemistry of the Romanian Academy, 41A Grigore Ghica Voda Alley, 700487 Iasi, Romania; duncaty@gmail.com; 2 ACADEMICHEM Kimya ARGE San. Tic. Ltd. Şti., Ege Üniversitesi Teknoloji Geliştirme Bölgesi, Izmir 35100, Turkey; 3 Leather Engineering Department, Faculty of Engineering, Ege University, Izmir 35100, Turkey; cigdem.kilicarislan@ege.edu.tr

**Keywords:** coating, epoxy, core-shell, morphology, reactive polymers, leather, finishing

## Abstract

The aim of the study is the synthesis and characterization of epoxy functional reactive polyacrylic latexes, e.g., poly (BA-co-MMA-co-AN-co-GMA) with core/shell and non-structured (random) particle morphologies. Additionally, their performance as binders and coating ability in leather finishing were studied. The epoxy functional polymers were synthesized via the seeded emulsion polymerization technique and the obtained latexes were characterized by means of particle size, zeta potential, FTIR, TEM, DSC, DMTA, and TGA. The results showed that the particle size and zeta potential values were very similar for both latexes, except core/shell latex had slightly higher particle size. DSC, TEM, and DMTA studies verified the successful synthesis of core/shell latex morphology. The copolymer films were elastic in nature and had low T_g_ values (−13 and −20 °C). The performance results showed some different behavior for core/shell and random copolymer coatings. The abrasion resistance of the leather finish with random copolymer as binder exhibited slightly better values, especially in wet conditions. On the other hand, the leathers finished with core/shell binder showed better performance in flexing endurance and the water spotting test.

## 1. Introduction

Waterborne acrylic polymers are widely used in paints, leather/textile finishes, paper, and wood coatings, etc., due to advantages such as ageing resistance, light stability, good pigment binding ability, good adhesion to various types of substrates, ease of application and their lower cost in comparison with other systems, e.g., polyurethanes [1,2,3]. However, they show low chemical and water resistance, reduced weathering stability, and insufficient mechanical and abrasion resistance [4,5,6]. To improve the coating performance of acrylic emulsions, they are usually combined with external crosslinkers such as isocyanates, polycarbodiimides, polyaziridines, and polyoxazolines, which react with the hydroxyl, carboxyl, or amine groups introduced in the acrylic polymer backbone [7,8,9,10]. The final crosslinked films give satisfactory mechanical and chemical resistance. However, these crosslinkers usually have toxic characteristics, and thus, they are harmful to human health and the environment. Moreover, the rate and amount of crosslinking must be well controlled during film formation, otherwise the elasticity of the coating can decrease significantly due to excessive crosslinking. The self-life of the dispersions in the presence of crosslinkers is also very short and the remaining mixtures after the application cannot be stored for a long time. Therefore, safer crosslinking options without the addition of toxic external crosslinking agents are desired for waterborne acrylic coating systems.

Acrylic polymer dispersions bearing reactive epoxy groups are an important class of functional polymers that are used in various industrial applications such as paints, adhesives, coatings, sealants, etc. [2,11,12,13]. The oxirane ring of the epoxy group can provide a wide range of modifications due to its reactivity with carboxyl, hydroxyl, and amine groups present in the coating and substrate. Glycidyl methacrylate is a vinyl acrylic monomer containing an epoxy group that can be copolymerized with conventional acrylic monomers to obtain epoxy-functional latexes [14]. The epoxy group on the polymer backbone can further be used as crosslinking sites to improve the mechanical, properties, water resistance, chemical resistance, and adhesion properties of latexes especially for coating purposes [15,16,17]. These coating systems are much safer to use in terms of environmental and health issues in comparison to the other external crosslinking polymeric systems. The coating performance of these reactive latexes and/or films is dependent on the number of epoxy groups, their distribution on the polymer, the size and stability of the particles, etc. Usually, partially cross-linked acrylic coatings can show better mechanical and chemical resistance. Therefore, the crosslinking degree must be well controlled during the film formation, especially for the coatings used for flexible surfaces such as leather and textiles.

Composite latex particles have been of great interest in the last decade due to their technology that allows us to create particle morphologies, giving us the possibility to combine different polymer properties [18,19,20,21,22]. This kind of particle morphology is often called as core-shell achieved by multi-stage emulsion polymerization, where a monomer composition is polymerized to form a core and another monomer composition is post-polymerized over the core-seed to form the shell. By selecting different monomer compositions for the core and shell phases, it is possible to obtain final polymer properties that are difficult to achieve by blending of two polymers or with random copolymerization. For instance, the good blocking and abrasion resistance of a coating is usually obtained by polymers having high T_g_ values. However, the use of high T_g_ polymers is limited due to their high minimum film-forming temperature (MFFT) and brittleness, especially for flexible coatings. On the other hand, with core-shell technology, it may be possible to synthesize elastic coatings with improved block and abrasion resistance [23]. The morphologies can be varied by changing the nature of core and shell phases, their ratios, or the number of layers. Even contrary properties, such as inorganic–organic, soft–hard, hydrophobic–hydrophilic, and low–high refractive indexes, can be combined to increase the functionality of the final polymer.

There are studies that use epoxy functionality in the preparation of core-shell latexes for coating purposes [14,24,25,26,27,28]. However, most of these studies include the use of pre-synthesized or commercial epoxy resin as a core or shell phase to prepare composite latexes, and there are few studies using epoxy functional monomer in the shell phase. It was proposed that using epoxy functional glycidyl methacrylate monomer in shell composition as a hard and reactive phase over a soft core polyacrylic latex can give improvements in overall performance of the latexes. The presence of reactive sites on the outer shell of the particles could lead to more controlled partial crosslinking, improving the resistance properties while the core remains soft to facilitate the adhesion and elasticity of the coating. Present study refers to the synthesis, characterization and preparation of a coating formulation based on poly(butylacrylate-co-methylmethacrylate-co-acrylonitrile-co-glycidyl methacrylate) by emulsion radical polymerization. The novelty aspect of the present manuscript is to contribute to the synthesis of latex binders with different morphologies (i.e., statistic and core-shell) and to investigate the effect of morphology on coating performance in a specific industrial application. Working with an epoxy-based monomer represents a challenge, as polymerization without affecting the epoxy ring is difficult. The epoxy ring must remain intact so that the newly formed polymers can be activated when applied in the real environment as result of the temperature action of the hot press, giving the finished leather unique properties. Both copolymer latexes with structured and non-structured morphologies were characterized and applied on leather as coating binder.

## 2. Materials and Methods

### 2.1. Materials

The monomers, butyl acrylate (BA, ≥99.0%), methyl methacrylate (MMA, ≥98.5%), glycidyl methacrylate (GMA, ≥97%), and acrylonitrile (AN, ≥99.0%) were purchased from Sigma-Aldrich, Schnelldorf, Germany and used in emulsion copolymerization. Sodium dodecyl benzene sulfonate (SDBS, Sigma-Aldrich, Steinheim, Germany), sodium bicarbonate (NaHCO_3_, Sigma-Aldrich, Steinheim, Germany), potassium persulfate (KPS, Sigma-Aldrich, Steinheim, Germany) were used as an emulsifier, a buffer agent and an initiator, respectively. All chemicals were used as received without any further purification. Ultrapure water was used as a solvent to perform the reactions. The syntheses took place in a four-neck round-bottom 250 mL glass reactor equipped with a condenser, nitrogen inlet, monomer dropping funnel, and initiator solution dropping funnel. For the reactions, a Heidolph (Schwabach, Germany) magnetic stirrer with a temperature control probe was used to heat the reactor in an oil bath.

Cattle crust leather suitable for footwear, gifted from Sepiciler Co. (İzmir, Turkey), was used for finishing applications. The other necessary finishing chemicals, combined with the synthesized copolymer emulsions such as wax emulsion, black pigment, nitrocellulose lacquer emulsion, and silicon-based touch modifier, were obtained from Stahl Co. (Waalwijk, The Netherlands).

### 2.2. Methods

#### 2.2.1. Synthesis of Acrylic Copolymer Emulsions

Two acrylic copolymer emulsions were synthesized via seeded emulsion polymerization with different structures of random and composite particles. Both emulsions consisted of the same components in the same amounts except for the preparation procedures. The experimental set-up used for polymerization was described in Table 1. The random copolymer emulsion (E1) was prepared according to the following procedure: given amounts of SDBS, NaHCO_3_, and 1/3 of KPS were dissolved in 70 g water and mixed in the reactor. A quarter (by volume) of the monomer mixture composed of BA/MMA/AN/GMA was added into the reactor and mixed at high speed (800 rpm) to prepare the pre-emulsion and purged with 10 mL·min^−1^ N_2_ flow for 30 min to remove the dissolved oxygen from the system. The reactor was then lowered into the oil-bath preheated to 80 °C and kept for 45 min at a 300 rpm mixing speed to prepare the seed latex. The remaining monomer feed and initiator solution were dropped into the reactor within 90 min. The reaction was kept for an additional 2 h to complete the polymerization.

**Scheme 1:**
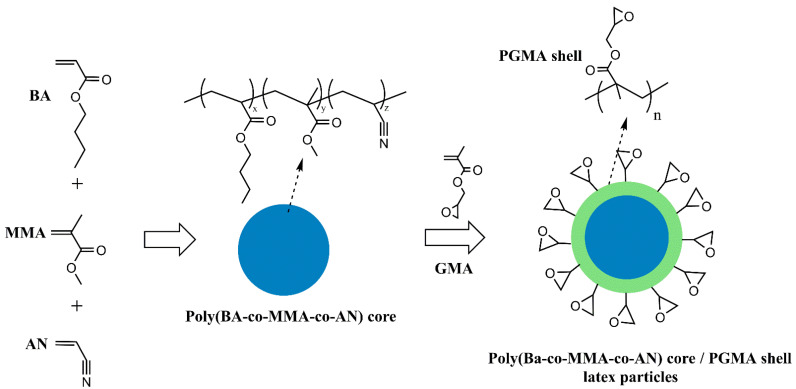
A representative illustration explaining the synthesis of poly(BA-co-MMA-co-AN) core/poly(GMA) shell latex particles.

The preparation of core/shell latex (E2) was done similarly, except for the composition of seed and monomer feeds. A diagram for the synthesis of the core-shell latex was presented in Scheme 1. In the polymerization procedure, SDBS, NaHCO_3_, and 1/4 of KPS were dissolved in 65 g water and added to the reactor1/3 of the core monomer mixture composed of BA/MMA/AN was introduced in the system, mixed at 800 rpm, purged with 10 mL·min^−1^ N_2_ flow for 30 min and reacted for 45 min at 80 °C at a mixing speed of 300 rpm to form the seed latex. Then the remaining monomer mixture of the core part was dropped into the system in parallel with 1/2 of KPS solution for 60 min and kept for an additional 60 min for completion of the reaction for the core-phase. GMA, as the shell phase monomer, was then added dropwise in the reactor within 30 min, in parallel with the remaining KPS solution. After the dropping was finished, the reaction was kept for 90 min to obtain the core-shell acrylic latex. At the end of both reactions, acrylic latex was obtained with a total solid content of 25 wt%. The nitrogen flow was kept constant at 10 mL·min^−1^ throughout all the reactions, and the reaction additives dropped in the system (monomers and KPS solutions) were previously purged with N_2_ to remove the dissolved oxygen. The monomer conversion ratios of both systems have been monitored gravimetrically throughout the polymerizations and found to be over 99% at the end of the reactions.

#### 2.2.2. Application of Acrylic Latexes in Leather Finishing

Leather finishing can be described as a process to improve the physical, mechanical, and aesthetic properties of the surface of leather products. Among the many factors, such as the type and quality of the leather, the chemical products, formulation employed, operating systems, the main factor effecting the appearance and performance of the leather surface is usually the coating composition. A great variety of chemicals are used in leather finishing processes. Among these chemicals, colorant agents (pigments and aniline dyes), synthetic binders (acrylates, polyurethanes, etc.), waxes, oils, lacquers (nitrocellulose, polyurethanes, caseins), fillers, dullers, feel modifiers, solvents, crosslinkers, etc. can be mentioned. The major constituent affecting the performance of the finishing layer is the binders, which are used to bind the other chemicals together and to the leather. Therefore, the synthesized reactive acrylic emulsions were used as binder in leather finishing application to assess their coating performance. The formulation of the finishing mixture was kept simple to evaluate the performance of the latexes, and it consisted of pigmented basecoat layers and a topcoat layer (Table 2). The coating layers were applied by hand spraying, followed by drying and hot plating at given intervals.

## 3. Characterization Methods

### 3.1. Particle Size and Zeta Potential Analysis

The average particle size, particle size distributions, and zeta potential of the nanocomposite latexes were measured by the NanoZS model (Malvern Instruments UK) zetasizer instrument. The samples were prepared by diluting the latexes 5000 times with distilled water.

### 3.2. Fourier Transform Infrared Spectroscopy (FTIR) Analysis

The structure was confirmed by FT-IR spectra of the copolymer films, which were recorded with a Perkin-Elmer Spectrum-100 ATR-FTIR instrument by scanning in the range of 600–4000 cm^−1^.

### 3.3. Differential Scanning Calorimetry (DSC)

DSC analysis was performed on copolymer films using a Perkin Elmer Diamond DSC instrument at a heating rate of 10 °C/min under N_2_ atmosphere from −70 to 250 °C.

### 3.4. Thermogravimetric Analysis (TGA)

Thermal decomposition behavior of the copolymers was assessed by using a TA Instruments Q600 model TGA device in the range of 30–700 °C with a heating rate of 10 °C/min under N_2_ atmosphere.

### 3.5. Dynamic Mechanical Thermal Analysis (DMTA)

Dynamic mechanical measurements were carried out using an Anton Paar MCR 301 Dynamic Mechanical Analyzer (Graz, Austria) equipped with dynamic mechanical analysis accessories at a constant frequency of 1 Hz, in the temperature range from −60 to 110 °C, under N_2_ atmosphere, with a heating rate of 3 °C/min. The films for the DMTA analysis were cut into rectangular specimens with a length of 10 cm and fixed between the oscillating and the fix parts of clamping device. All measurements were performed in extension mode with a constant deformation strain of 0.1% and the storage modulus E′, the loss modulus E″, and the value of the tan δ (tan δ = E″/E′) were determined for all temperature range.

### 3.6. Transmission Electron Microscopic (TEM)

The morphology of the latex particles was investigated by transmission electron microscopy (using a Carl Zeiss 300VP FE-SEM device. For TEM investigation, latex samples were diluted with water, mixed with 2.0 wt% aqueous solution of phosphotungstic acid, placed on a carbon-copper grid by a micropipette, and dried at room temperature. The scale of the magnification is given in the images.

### 3.7. Scanning Electron Microscopy (SEM)

The imaging of the finish layer of the leather samples coated with latex was performed by the Hitachi TM-1000 Table-Top scanning electron microscope. The samples (~5 × 1 mm) were cut from the cross-section region of the leathers and were placed on the device plate by using double-sided adhesive tape. The magnification levels are given on the images.

### 3.8. Physical Tests Performed on the Finished Leathers

To evaluate the performance of the copolymer latexes as leather coating binders some performance tests were carried out on the finished leathers. The leathers to be tested by physical methods were first conditioned according to the standard of EN ISO 2419 and the sampling was carried out according to EN ISO 2418. The finished leathers was tested by physical standard methods of: flexing endurance [29]; color fastness of leather to “To” and “Fro” rubbing [30] and color fastness to water spotting [31]. The evaluation of all the tests related to color change was done according to the gray scale standard [32,33], which gives a rating between 1 and 5 (5: means no color change, and 1: means failure).

## 4. Results and Discussion

### 4.1. Particle Size and Morphology

Latex stability is an important issue, especially for latexes comprising GMA due to the high reactivity nature of the oxirane ring. Therefore, particle gelation and coagulum problems can be encountered frequently during emulsion polymerization [28]. To ensure the particle stability and reduced reactivity of epoxy groups, the polymerization was carried out in a controlled manner by slow monomer feeding in the presence of NaHCO_3_ buffer and a final solid content of 25%. Both random and core-shell structured latexes were obtained coagulum free and with high conversion rates (>99%) at the end of the reactions.

**Figure 1:**
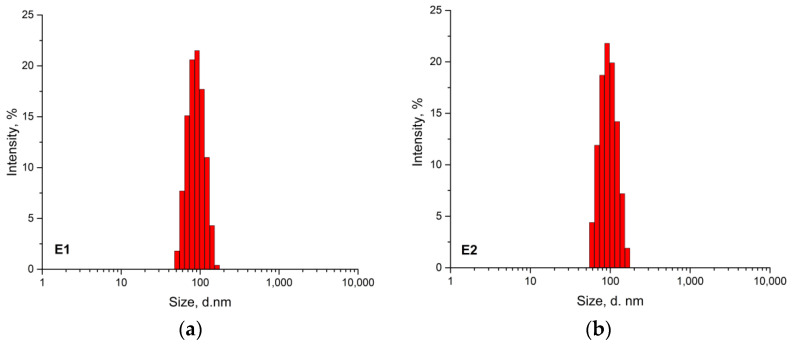
The particle size distribution of the latexes (**a**) E1-random copolymer latex; (**b**) E2-composite copolymer latex.

The particle size distribution of both latexes obtained by dynamic light scattering is given in Figure 1 and Z-average particle diameter, polydispersity index (PDI), and zeta potential values were summarized in Table 3. The obtained results showed that both latexes had fine particle size with an average particle size of 85.5 nm for E1 and 91.7 nm for E2; both latexes had very narrow size distributions with low PDI values (~0.04). The zeta potential measurements showed that Latex E1 had a value of −42.2 mV while E2 latex had a slightly decreased value of −46.7 mV indicating that both latexes were electrostatically very stable (Table 3).

**Figure 2:**
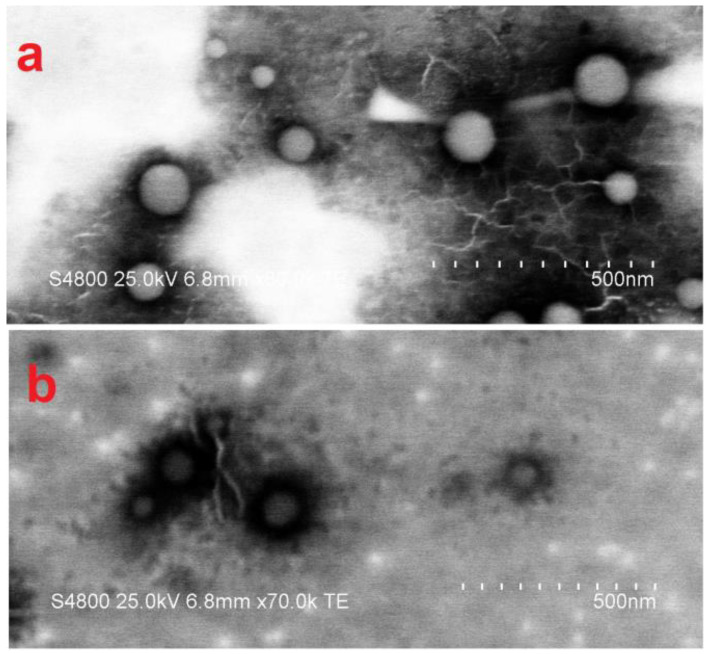
TEM images of the latex particles: (**a**) Latex E1, (**b**) Latex E2.

Figure 2 shows the TEM images obtained from the latexes. From both images, the particle sizes could be calculated between 50–120 nm, which was in accordance with data obtained from light scattering measurements. The upper image (Figure 2a) belongs to the random copolymer latex where whole particles can be clearly seen as white spheres. The other image (Figure 2b) shows the core-shell latex particles. In the image, a difference in contrast of core and shell parts can be observed. The darker regions around the particles were possibly due to the interaction of staining agent with the shell phase monomer more intensively. Overall results indicated that stable latex particles with different particle morphologies were obtained.

### 4.2. FTIR Analysis

**Figure 3:**
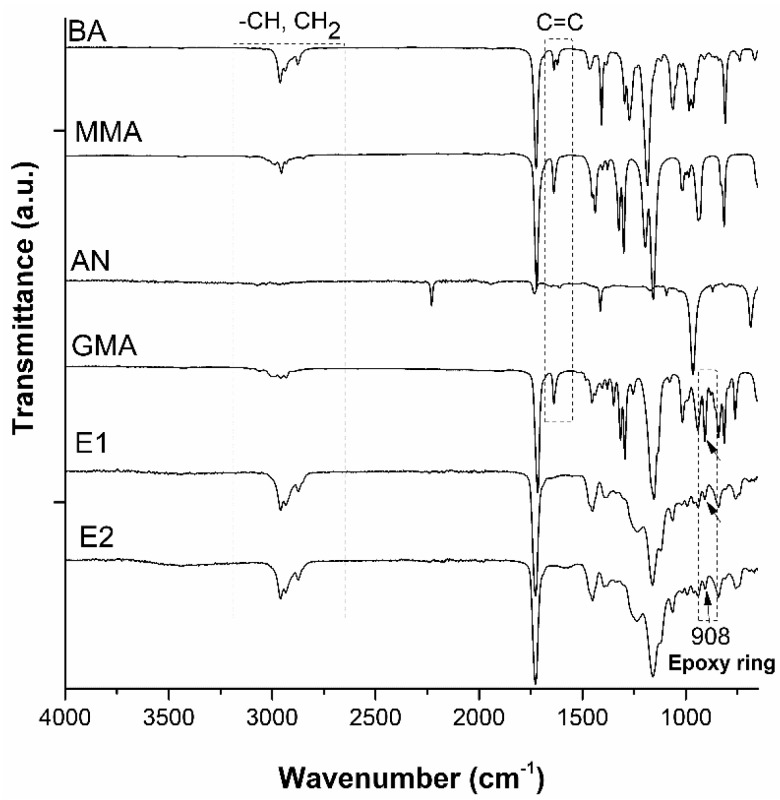
ATR—FTIR spectra of the synthesized latexes (E1 and E2) and pure monomers.

The substance identity was confirmed by the ATR-FT-IR spectra of the obtained latexes and the pure monomers. The spectral characteristics were shown in Figure 3 and the main peaks were assigned to the specific vibration bands of the functional groups and the modifications were evaluated by comparing the spectra of polymers with monomers.

From the spectra of both copolymers, it was observed identical absorption characteristics where *asym* and *sym* stretching of -CH groups of CH_2_ and -CH_3_ at 2959, 2934, and 2874 cm^−1^, respectively, C=O stretching at 1727 cm^−1^, -CH_2_ and -CH_3_ deformation at 1452 and 1387 cm^−1^, C-O-C stretching at 1160 cm^−1^. The absence of the vibration peaks around 1608–1634 cm^−1^ assigned to the vinyl groups of acrylates for both latexes marked the success of the polymerization. The specific absorption peak of the epoxy ring was observed clearly at 905 cm^−1^, which indicated that the attachment of these reactive groups was achieved without opening the oxirane ring, leading to reactive epoxy-based polymers.

### 4.3. Thermal Characteristics of the Copolymers

**Figure 4:**
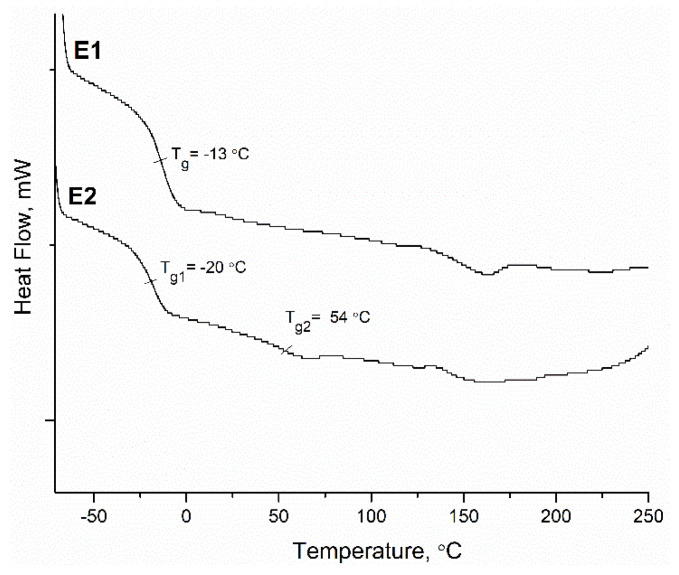
DSC thermograms of random copolymer latex (E1) and composite core—shell acrylic latex (E2).

**Figure 5:**
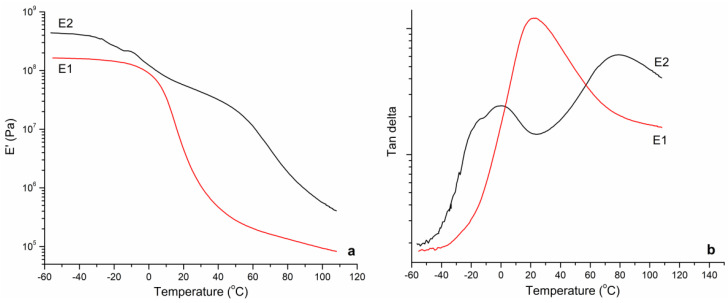
The elastic modulus (**a**) and Tan—D curves (**b**) of the copolymer films obtained from DMTA.

**Figure 6:**
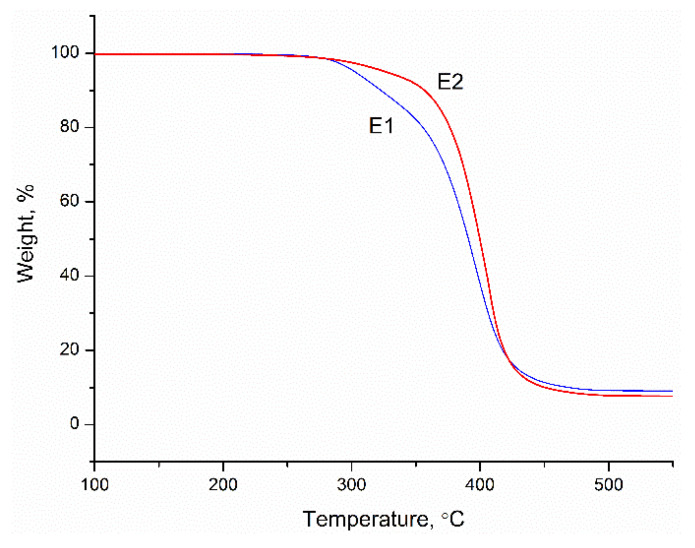
TGA curves of the copolymers.

Thermal characteristics of the synthesized polymeric latexes were determined by DSC, DMTA, and TGA, and the results are presented in Figure 4, Figure 5 and Figure 6.

Figure 4 shows the DSC thermograms obtained from both latex films. The thermogram of the random copolymer (E1) showed one large and clear phase transition due to a T_g_ at −13 °C, which was comparable to the theoretical T_g_ value (−18, 7 °C) calculated by the Fox equation. On the other hand, from the thermogram of E2, two different phase transitions could be observed. The first T_g_ value of soft core was determined at −20 °C and second T_g_ of poly(glycidyl methacrylate) shell at 55 °C being comparable to theoretical T_g_ values (T_g1_ = 29 °C T_g2_ = 61 °C). Because the ratio of the shell part was relatively low (15 wt%), the transition of shell phase observed at DSC analysis was small. It is known that the different segments of copolymers can be observed more clearly from the thermo-mechanical behavior. Therefore, DMTA analysis was performed on the copolymer films. The elastic modulus (E′) and Tan D curves of the copolymer films as a function of temperature obtained from DMTA are given in Figure 5. From E′ curves, the glass transition regions of the copolymers were very clear. From the peaks of the Tan D curves, the only glass transition of E1 copolymer was observed at 20 C whereas the first transition of soft core part of E2 copolymer was at −5 °C and second transition due to the harder shell part was around 80 °C. Moreover, the elastic modulus of the core-shell polymer was much higher than random copolymer for all temperature ranges. For instance, the E′ value of E1 was 1.86 MPa at room temperature and for E2 it was found to be much higher at 49.1 MPa.

The thermal degradation behavior of the copolymers was assessed with TGA and the results are given in Figure 6. From the curves, we can see that the thermal degradation behavior was also affected by the morphology of the particles. The results showed that the degradation process of E1 started earlier than E2 and degraded faster almost till the end of the process. This also indicated that harder segments of the shell phase led to increased thermal degradation stability of the copolymer films, similar to mechanical properties.

### 4.4. Coating Performance of the Copolymer Latexes

The synthesized latexes were applied on shoe upper leathers as a binder in a finishing formulation (Table 2). The coats were applied with light sprays on leathers by hand spraying gun with an air pressure of 3 atm. After applying each coat, the leather was dried in a drying tunnel at 80 °C for 3 min. At given intervals (Table 2), the coated leathers were hot pressed at 80 °C for staining the coats and increasing the adhesion of the layers.

After the application, all leathers showed a uniformly colored and glossy appearance with a natural look. To promote the crosslinking reaction of the epoxy groups in the coatings, the leathers were kept at 50 °C for 24 h and subsequently rested for 2 weeks prior to the performance tests.

**Figure 7:**
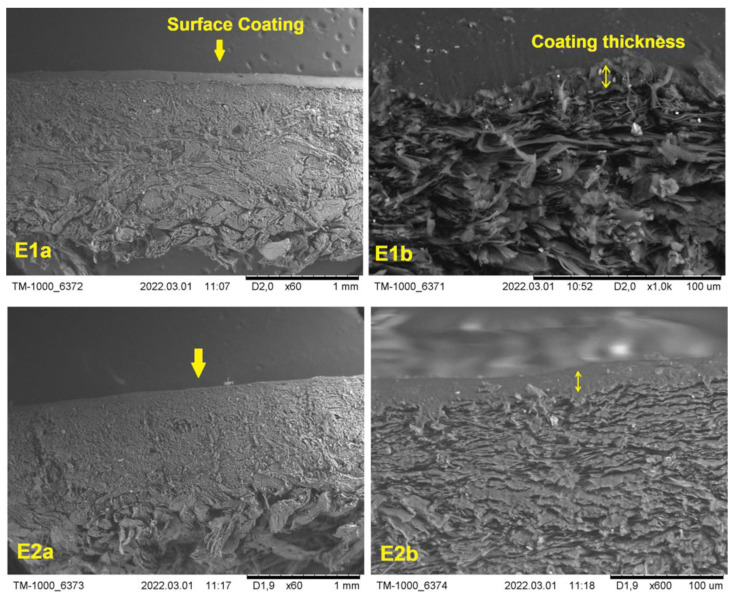
The SEM images of the cross-sections of finished leathers at different magnifications.

The SEM images of the cross sections of finished leathers are given in Figure 7. In the images, the finishing layer applied to the leathers is shown at different magnifications. It can be observed that the thickness of each finishing layer, in respect to the thickness of the overall cross section, was very small. At higher magnifications, the thickness of the finishing layer could be calculated within the range of 10–20 µm for all samples. This verified that the coating film was thin enough to be able to keep the natural grain appearance of the leather, since the appearance is usually lost when the finishing layer thickness is more than 100 µm.

One of the most important and required tests for every colored/dyed leather is rubbing fastness. This test is used to determine the degree of color transfer from the surface of the finished leather to a felt that moves over the leather surface to create an abrasion action. The degree of coloring of the felt and deterioration of the leather surface are evaluated, and results are given in accordance with the gray scale rating. The results equivalent to 3 or higher are accepted as satisfactory, and 5 is the maximum value. The results obtained from the rubbing test are summarized in Table 4. From the results, it was observed that both samples had very good dry rubbing fastness values, even at 250 rubbing cycles. The wet rubbing fastness levels of the leathers were also satisfactory, even after 50× rubbing. However, comparing random copolymer (E1) with composite latex (E2), it was determined that the random copolymer gave slightly better results for wet rubbing fastness. This could be due to the more evenly distributed crosslinking sites provided by the epoxy groups of the random copolymer within the coating that resulted in better resistance to abrasion in wet conditions. The positive effect of the uniform cross-link distribution on mechanical properties was also reported by other researchers [34].

The results obtained from the flexing endurance test are given in Table 5. The test simulates the bending of leather products during daily use and evaluates the cracking of the film at the bending region. The leathers coated with the copolymers underwent movement cycles of 50,000 and 100,000 times, and at each interval the leather finish was examined for any damage (greying, surface cracks, loss of adhesion etc.). The results showed that after 50,000 cycles no visible damage was observed for both samples except slight wrinkles at the bending region. After 100,000 cycles, the wrinkles were much deeper and small cracks were observed for leathers finished with random copolymer (E1), whereas only light wrinkles were observed for the other leathers. This result was probably because of the softer nature of the core phase of composite latex (E2) that provided better flexibility to the final coating than random copolymer.

**Figure 8:**
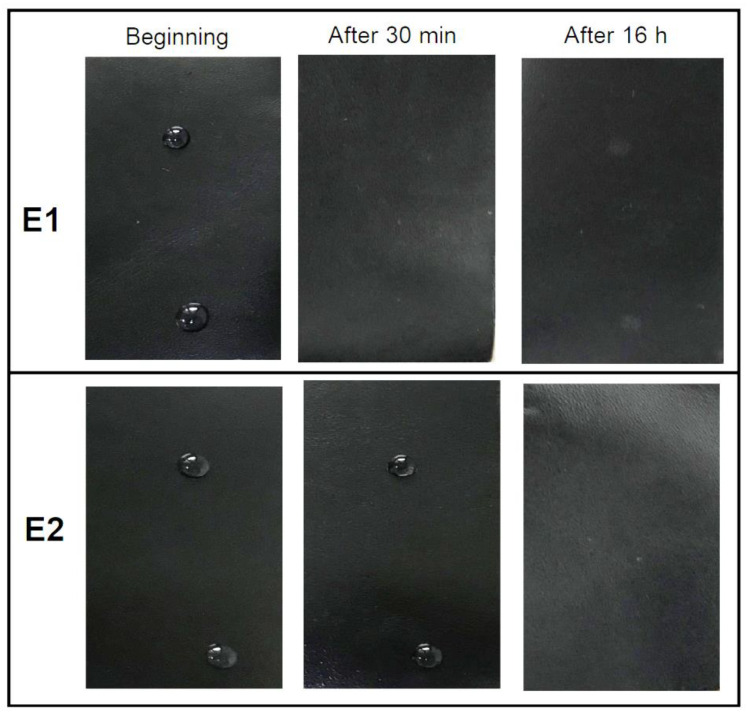
The images taken at the beginning of, 30 min and 16 h after placing the water drops on the leather surface.

Another test that gives information about the water resistance of the leather surface is the color fastness of leather to water spotting. During the test, two drops of distilled water were placed on the surface of the leather; after 30 min, the surplus of water was removed mechanically with filter paper from one of the drops, and the physical effects were observed, if any. The other drop was allowed to evaporate overnight, and any change in color of the leather was assessed with the standard gray scale. The results were given in Table 6 and the images of the leathers at each interval were presented in Figure 8. After 30 min, the leather E1 absorbed all the water drops, whereas both drops remained on the surface of leather E2. At the end of the 16 h period, the water drops were completely absorbed and/or evaporated for all samples; there remained a lighter color spot on the leather E1 and no spotting on leather E2.

The results indicated that the leather surface coated with core-shell structured latex had more water repellent behavior than that with random copolymer. This phenomenon was could be due to the pronounced hydrophobic nature of the soft core and crosslinked shell phase, resulting in a more water repellent behavior of the final coating and/or also could be related with the micro-roughness of the surface which needs further morphological investigations.

The general evaluation of the physical test results exhibited that the leathers coated with both latexes had good performance levels in comparison to the acceptable quality standards defined by UNIDO for the leather and footwear industry [35].

## 5. Conclusions

Epoxy functional reactive polyacrylic latexes with core/shell and non-structured (random) particle morphologies have been synthesized successfully. Even though the polymerization of epoxy-based monomers arises stability issues, both latexes with a GMA content of 15 wt% were coagulum free, electrostatically stable and with a small particle size. Due to the reactive hard-shell phase of the copolymer, the elastic modulus (E′) of the composite latex, which is an indication of the strength to resist when elastically deformed upon a stress application, was much higher than random copolymer, especially within the temperature range of 20–60 °C. Similarly, TGA results also showed improved thermal stability for the composite latex copolymer. After the leather finishing application, it was shown that the random copolymer had slightly better abrasion resistance, possibly due to the more homogenously distributed crosslinked sites of the final coating film, but the leathers finished with composite latex exhibited a better performance in the flexing endurance and water spotting tests. The main obtained outcome is that although the composition of copolymers was identical, the particle morphology had a significant effect on both polymer properties and application performance. This effect is correlated with desired properties for the final products, particularly in better coating ability, penetration, and thus mechanical, thermal, and coating performance. The synthesized latexes with self-crosslinking ability in both cases had good finishing performance values, taking into account general requirements for leather products [35]. In respect to the fact that most of the commercial leather finishing systems use toxic external crosslinkers when high performance values are needed, present latexes provide an advantage that no further external crosslinker is necessary.

## Figures and Tables

**Table 1:** The composition of acrylic latexes.

Ingredients	E1 (Parts)		E2 (Parts)		
	Seed	Feed	Seed	Core-Feed	Shell-Feed
BA	4.00	12.25	5.45	10.80	-
MMA	0.60	1.90	0.63	1.87	-
AN	0.60	1.90	0.63	1.87	-
GMA	0.90	2.85	-	-	3.75
SDBS	0.50	-	0.50	-	
NaHCO_3_	0.075	-	0.075	-	
KPS	0.017	0.033	0.0125	0.025	0.0125
Distilled water	70.00	5	65	5	5

**Table 2:** The finish formulation applied on the leathers.

Components	Application Steps		Descriptions
	Basecoat (I) (Parts)	Topcoat (II) (Parts)	
Water	40	20	Spray I × 3 times
Pigment	10		Hot plate 80 °C/100 bar
Wax	5		
Acrylic Latex	25		Spray I × 3 times
Isopropyl alcohol	0.5		
Aqueous NC Lacquer		10	Spray II × 1 time
Silicone Surface Modifier		0.5	Hot plate 80 °C/70 bar

**Table 3:** Average particle size diameters, polydispersity index and zeta potential values of the latexes.

Emulsion	Z-Average Diameter (nm)	Polydispersity Index (PDI)	Zeta Potential (mV)
E1	85.5	0.040	−42.2
E2	91.7	0.043	−46.7

**Table 4:** Rubbing fastness properties of leather samples coated with copolymer latexes.

Samples	Evaluated Parts	Gray Scale Values			
		Dry		Wet	
		50 Cycles	250 Cycles	25 Cycles	50 Cycles
E1	Leather	5	5	5	5
	Felt	5	5	5	4/5
E2	Leather	5	5	4/5	4
	Felt	5	5	4/5	3/4

**Table 5:** Flex resistance of leather samples.

Samples	50,000 Cycles	100,000 Cycles
E1	Light wrinkles	Deep wrinkles and small cracks
E2	Very light wrinkles	Light wrinkles

**Table 6:** Color fastness of leather samples to water spotting after 30 min and 16 h.

	30 min		16 h	
Leathers	Observation	Gray Scale Value	Observation	Gray Scale Value
E1	Completely absorbed by leather, remained a visible spot after drying	4	Completely absorbed/evaporated, remained a visible spot	3/4
E2	The water drop remained with its initial form, no spot after drying	5	Completely absorbed/evaporated, no spot	5

## Data Availability

Data sharing is not applicable to this article.
